# Renalase protects against podocyte injury by inhibiting oxidative stress and apoptosis in diabetic nephropathy

**DOI:** 10.1515/biol-2022-0940

**Published:** 2024-11-22

**Authors:** Yiru Wu, Yiduo Feng, Yue Yu, Yu Bai, Zongli Diao, Wenhu Liu

**Affiliations:** Department of Nephrology, Faculty of Kidney Diseases, Beijing Friendship Hospital, Capital Medical University, Beijing, 100050, People’s Republic of China; Department of Nephrology, Beijing Tiantan Hospital, Capital Medical University, Beijing, 100070, People’s Republic of China; Department of Nephrology, Liangxiang Hospital, Fangshan District, Beijing, 102401, People’s Republic of China

**Keywords:** apoptosis, diabetic nephropathy, oxidative stress, podocyte, Renalase

## Abstract

Diabetic nephropathy (DN) presents a significant public health challenge due to its high rate of incidence and severe health consequences. Renalase has been identified as having renal-protective properties. A key contributor to albuminuria in DN patients is podocyte loss. The function of Renalase in DN in relation to podocyte activity needs to be explored further. In this study, we assessed the therapeutic efficacy of Renalase by monitoring changes in urine protein levels and podocyte health in db/db mice. We also induced hyperglycemia (HG) to stimulate podocyte clone 5 (MPC5) cells to create a model of podocyte loss in DN. Through co-culturing these cells with Renalase or H_2_O_2_, we investigated the process by which Renalase prevents podocyte loss *in vitro*. In db/db mice, Renalase expression was significantly reduced, and adenoviral-mediated Renalase expression markedly alleviated DN symptoms and proteinuria. Furthermore, podocytopathy in db/db mice was significantly mitigated. *In vitro*, Renalase improved the expression of podocyte marker proteins, podocin, and nephrin, which are reduced by HG, as well as decreased oxidative stress and restrained apoptosis. Our findings suggest that Renalase can mitigate DN by reducing proteinuria through podocyte protection, potentially by inhibiting oxidative stress and apoptosis. These data suggest that Renalase may serve as a novel therapeutic agent in suppressing DN.

## Graphical abstract

Annotation: The red line represents inhibition; Blue represents improvement

**Figure:**
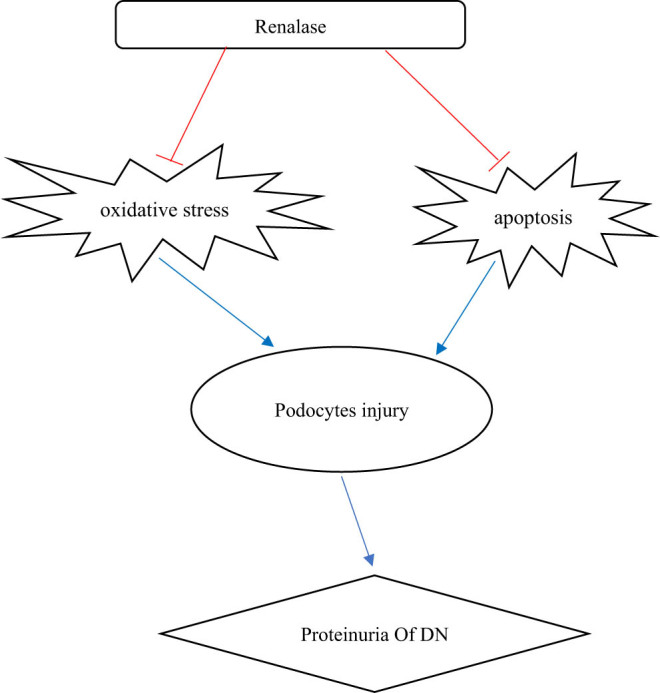

## 1 Introduction

Diabetic nephropathy (DN), also known as diabetic kidney disease, is considered as one of the most severe microvascular complications associated with diabetes mellitus. It is characterized by a progressive increase in proteinuria [1,2]. Currently, there are no effective treatment strategies for DN in clinical practice. The typical approach primarily involves symptom treatment such as the use of renin-angiotensin system inhibitors to lower blood pressure, which are employed as first-line therapies for a variety of diseases. Additionally, efforts are made to control blood glucose levels and manage weight. However, these treatments have not proven to be reliable [3,4]. When DN progresses to end-stage renal disease (ESRD), patient survival relies on dialysis or kidney transplantation. Currently, DN is the leading cause of new ESRD cases worldwide, imposing a significant burden on both the economy and families [5,6].

Typically, the damage to podocytes in diabetic patients leads to the degradation of the glomerular filtration membrane, resulting in proteinuria and the advancement of DN [7,8]. Due to various genetic and systemic damage factors in DN, the changes in the internal environment can affect podocytes, resulting in the effacement of foot processes and eventually podocyte detachment, which leads to proteinuria and the development of glomerulosclerosis [9]. Considering the lack of effective measures to prevent podocyte damage, it is essential to further explore the molecular biology related to the various podocyte diseases to find possible therapeutic targets for DN [10].

Renalase, a flavoprotein with oxidoreductase activity, not only prevents kidney diseases [11] but also protects the heart [12,13]. Renalase exerts its cytoprotective effects through its plasma membrane receptors, rather than by metabolizing catecholamines [13,14]. Hence, Renalase, a kidney-derived cytokine, may regulate the function of cells though autocrine or paracrine signaling. Renalase has been shown to protect the kidney against acute kidney injury (AKI), contrast nephropathy [15], and DN [16]. Given the critical role of podocyte injury in the occurrence and development of DN, it is not clear whether Renalase can delay the progression of DN by alleviating podocyte injury. Building on our previous studies [17,18], Renalase has been investigated in this study for its potential to alleviate DN by reducing podocyte loss.

## 2 Materials and methods

### 2.1 Animal models

We sourced male C57BL/6J db/m normal and db/db diabetic mice from the Institute of Laboratory Animal Science. All mice were subjected to a 12 h light/dark cycle with unrestricted access to water for 1 week. Subsequently, they were divided into four groups (*n* = 6 each). Adenoviruses were purchased from Shanghai Genechem Co., Ltd. Each group received injections via the tail vein as follows: (1) db/m mice treated with 1.0 × 10^10^ plaque forming units (PFU) of control adenovirus (db/m + Ad-b-gal), (2) db/m mice treated with 1.0 × 10^10^ PFU of adenovirus-Renalase (db/m + Ad-Renalase), (3) db/db mice treated with 1.0 × 10^10^ PFU of control adenovirus (db/db + Ad-b-gal), and (4) db/db mice treated with 1.0 × 10^10^ PFU of adenovirus-Renalase (db/db + Ad-Renalase). All animals were euthanized at 19 weeks. Following euthanasia, kidneys and blood were collected for various analyses.


**Ethical approval:** The research related to animal use has been complied with all the relevant national regulations and institutional policies for the care and use of animals. The Animal Experiment Ethics Committee of Beijing Friendship Hospital approved the experimental protocol, adhering to the Guidelines for the Care and Use of Experimental Animals as published by the National Institutes of Health (project approval number: 20-2016, project approval date:2022-10-24).

### 2.2 Detection of proteinuria level

The urinary albumin to creatinine ratio was utilized to evaluate urinary protein levels. Each mouse was individually placed in a metabolic cage for 24 h to collect urine. The concentrations of urine albumin and creatinine were measured using an Enzyme-Linked Immunosorbent Assay according to the manufacturer’s instructions after the 24 h collection period.

### 2.3 Histopathological examination

Various microscopy techniques, including light microscopy, polarization microscopy, and transmission electron microscopy, were utilized for histopathological examination. For each mouse, three kidney sections were stained with hematoxylin–eosin (HE), periodic acid–Schiff (PAS), Masson’s trichrome (Masson), and Sirius Red (SR). Pathomorphological changes were observed with HE, PAS, and Masson stains using light microscopy, while changes with SR staining were examined using polarization microscopy. The severity of renal fibrotic lesions was quantified by calculating the percentage of MTS- and SR-positive areas using the Image Acquisition and Analysis Software LabWorks (Ultra-Violet Products, Cambridge, UK). For each sample, five randomly selected nonoverlapping fields were analyzed. Transmission electron microscopy was employed to observe morphological changes in podocytes.

### 2.4 Immunohistochemistry

Paraffin-embedded kidney tissue sections were incubated with primary antibodies (concentration: 1:200) that target nephrin (ab216341; Abcam), podocin (ab50339; Abcam), and Renalase (ab178700; Abcam) overnight at 4°C. Adhesion to secondary antibodies was detected using an ABC ELITE kit (Vector Laboratories, Burlingame, CA, USA) containing secondary antibodies. Sections that were stained with the secondary antibody alone served as negative controls. Quantification of immunohistochemistry was performed using Image-Pro Plus software (Media Cybernetics, Rockville, MD, USA). The optical density (IOD) was calculated as density (mean) × area; *e* density represents the concentration or intensity of the reaction-positive protein. The mean density (MOD) was calculated as IOD/(SUM × area).

### 2.5 Cell culture and treatments

Mouse podocyte clone 5 (MPC5), an immortalized mouse podocyte cell line, was acquired from BeNa Culture Collection (BNCC337685, Beijing, China). We cultured the cells in DMEM/low glucose medium (Glenview, Florida, USA) supplemented with 10% FBS (Gibco, Carlsbad, CA, USA) and recombinant IFN-γ (PeproTech, London, UK) at 33℃ to promote cell proliferation, and then without IFN-γ for differentiation for more than 7 days at 37℃.

Upon reaching approximately 70% confluence, the podocytes were maintained for 12 h in serum-free conditions before initiating treatments. The cells were divided into the following groups: (1) control group, where podocytes were cultured in 5 mmol/L glucose; (2) hyperglycemia (HG) group, where podocytes were cultured in 25 mmol/L glucose; and (3) HG + Renalase group, where podocytes were exposed to 25 mmol/L glucose with varying concentrations of Renalase (100, 500, 1,000 ng/mL). Subsequently, cells were collected for analyses. Additionally, MPC5 cells underwent similar conditions and were incubated with HG (25 mmol/L) in the absence or presence of H_2_O_2_ (500 µM/L) and Renalase (1,000 ng/mL) for 48 h to investigate its podocyte protection activity.

### 2.6 Western blot analysis

As previously described, we prepared whole cell lysates and kidney tissue homogenates for immunoblotting [17]. The primary antibodies used included anti-nephrin (ab216341; Abcam) and anti-podocin (ab50339; Abcam), with a dilution of 1:1,000.

### 2.7 Apoptosis measurements

Cells from the MPC5 line were counted and centrifuged at 1,200 rpm for 5 min at room temperature. The cells were then incubated with 195 µL of Annexin V-FITC binding solution and 5 µL of Annexin V-FITC for 10 min at room temperature. The Annexin V-FITC cell apoptosis detection kit (Beyotime Institute of Biotechnology, Haimen, China) was used to assess apoptosis.

### 2.8 Detection of oxidative stress

Malondialdehyde (MDA), a byproduct of lipid peroxidation of membrane polyunsaturated fatty acids, serves as an indicator of oxidative damage. Superoxide dismutase (SOD) is crucial for scavenging oxygen-free radicals in the body, protecting against oxidative damage to cells and facilitating the repair of damaged cells. Levels of MDA and SOD *in vitro* were measured using commercial kits according to the manufacturer’s protocols (Beyotime, Nantong, China) to assess the balance between oxidative stress and antioxidative responses.

### 2.9 Statistical analyses

Data are presented as mean ± standard deviation (SD). Statistical analyses were conducted using SPSS 17.0 software (IBM-SPSS, Armonk, NY, USA). Comparisons between groups were performed using one-way analysis of variance followed by the Student–Newman–Keuls test. A *p*-value <0.05 was considered statistically significant. Results from the animal studies are expressed as a percentage of the control metrics, representing the mean ± SD for five animals in each group. Cell experiment data were replicated three times in each group.

## 3 Results

### 3.1 Renalase ameliorates pathological changes of kidney in db/db mice

**Figure 1:**
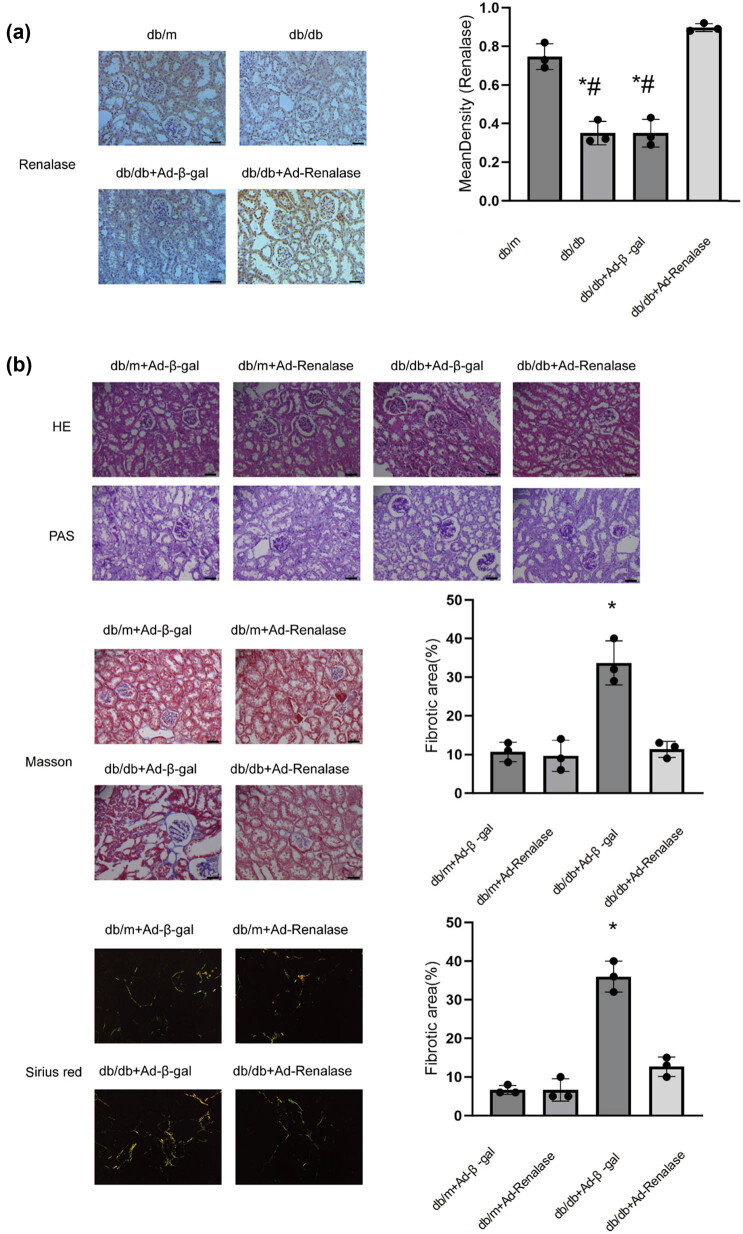
Role of Renalase on DN. (a) Expression of Renalase in mice. Immunohistochemistry showing that Renalase is mainly expressed in proximal renal tubules. In db/db mice its expression decreased significantly, but when injected with Renalase-overexpressed adenovirus into the tail vein, the down-regulated expression was corrected. *p < 0.05, compared with db/m group, ^#^ *p* < 0.05, compared with db/db + Ad-Renalase group. (b) Kidney sections from various groups were subjected to HE, PAS, Masson’s trichrome, and Sirius red staining. Renal fibrotic lesions (defined as the percentage of the MTS- and SR-positive fibrotic area) were quantified by computer-aided morphometric analyses. Representative micrographs showing Renalase ameliorated renal pathology injury, mainly hyperplasia of the mesangial matrix, glomerular hypertrophy, and renal interstitial fibrosis was alleviated. Magnification 40×; *p < 0.05, compared with db/m + Ad-β-gal group.

As previously described in Section 2, the expression of Renalase was increased through adenovirus-mediated gene delivery to investigate its relationship with DN. Renalase expression was found to be significantly reduced in db/db mice compared to db/m mice, a condition reversed upon adenoviral injection to overexpress Renalase (Figure 1a). This down-regulation of Renalase in DN mice may play a role in the onset and progression of the disease. Subsequently, we examined the pathological changes in the kidneys. In the db/m + Ad-β-gal and db/m + Ad-Renalase groups, the kidneys displayed no apparent fibrous tissues; the renal tubules were well-organized, and the basement membranes were smooth and orderly. Glomeruli appeared normal in size with no noticeable thickening of the basement membrane or hyperplasia of the mesangial matrix. In contrast, the db/db + Ad-β-gal group exhibited glomerular hypertrophy, thickening of the glomerular basement membrane, mesangial matrix hyperplasia, and deposition of renal interstitial fibrous tissue, which were all minimal in the db/db + Ad-Renalase group (Figure 1b). These findings indicate that Renalase can improve the pathological abnormalities in the kidneys of db/db mice.

### 3.2 Renalase relieved urinary protein level in db/db mice

**Figure 2:**
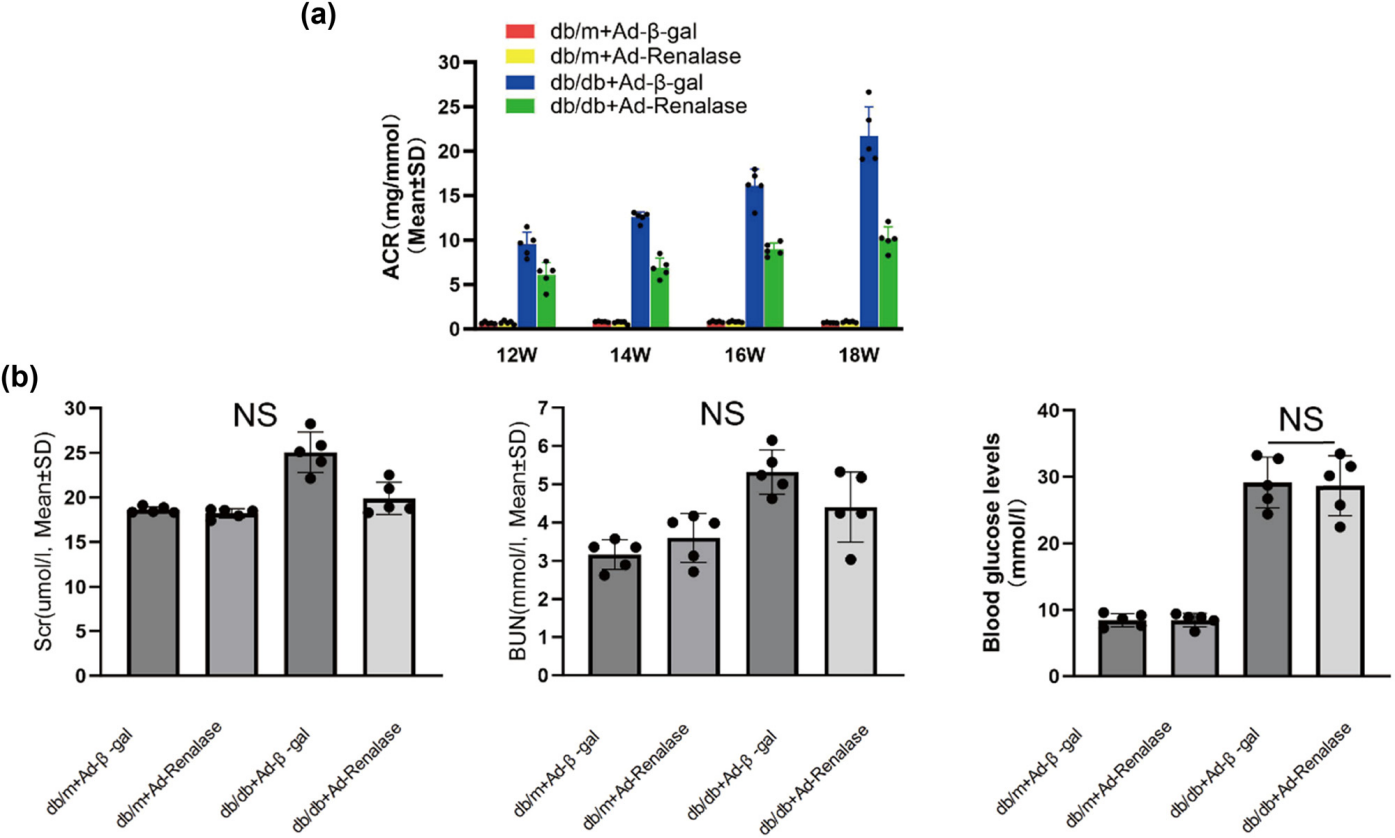
Urinary protein, Scr, BUN, and blood glucose levels in each group. (a) Proteinuria increased significantly in db/db + Ad-β-gal group versus db/m + Ad-β-gal and db/m + Ad-Renalase group; when adenovirus–Renalase was injected through tail vein, the level of urinary protein declined obviously. (b) Scr and BUN levels increased in db/db + Ad-β-gal group, but the difference was not statistically significant. In db/db + Ad-Renalase group, this increased trend disappeared. There was no significant difference in blood glucose levels between the db/db + Ad-β-gal and db/db + Ad-Renalase groups. All results are the mean ± SD of five animals per group. NS: not statistically significant between groups.

Urinary protein is a critical factor in the development and progression of DN. Therefore, we evaluated the effect of Renalase on urinary protein levels to explore its kidney protective activity in DN. Compared to the db/m + Ad-β-gal and db/m + Ad-Renalase groups, urinary protein excretion was significantly elevated in the db/db + Ad-β-gal group. Conversely, when Renalase expression was upregulated, there was a marked decrease in urinary protein excretion in the db/db + Ad-Renalase group (Figure 2a). These results suggest that Renalase alleviates urinary protein levels in db/db mice, potentially slowing the progression of DN. Additionally, serum creatinine (Scr) and urea nitrogen (BUN) levels were measured. While there was a slight increase in Scr and BUN levels in the db/db + Ad-β-gal group, the differences were not statistically significant. Moreover, this increasing trend was absent in the db/db + Ad-Renalase group (Figure 2b). We have also demonstrated that there was no significant difference in blood glucose levels between db/db + Ad-β-gal and db/db + Ad-Renalase groups, indicating that Renalase does not have a lowering effect on blood glucose (Figure 2b). That is, the impact of Renalase on reducing urinary protein in DN mice was not associated with blood glucose levels.

### 3.3 Improvement of DN by Renalase may be related to the reduction of podocyte damage in db/db mice

**Figure 3:**
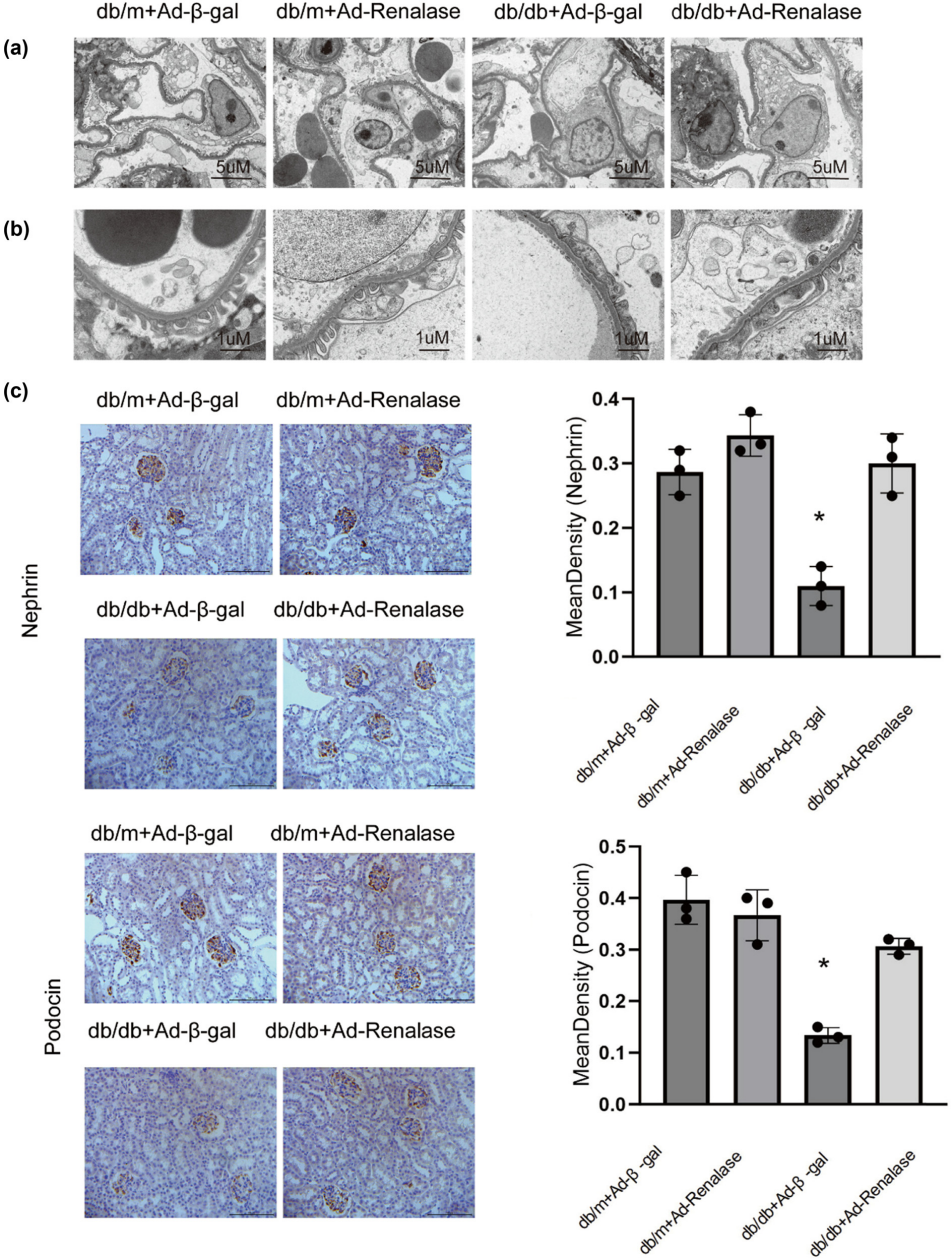
Changes of podocyte and its marker protein in each group. (a and b) Podocytosis under electron microscopy. It shows there were fusions and disruption of foot processes of podocytes in db/db + Ad-β-gal group versus db/m + Ad-β-gal and db/m + Ad-Renalase group. When Renalase adenovirus was injected into the tail vein in db/db + Ad-Renalase group, the appeal lesions markedly improved. (c) Expression of nephrin and podocin in podocytes. Immunohistochemistry showed that nephrin and podocin deposition reduced in db/db + Ad-β-gal group, while the change was significantly mitigated in db/db + Ad-Renalase group. Magnification 40×; *p < 0.05, compared with db/m + Ad-β-gal group.

Renalase has been shown to impede the progression of DN and reduce proteinuria. To understand the underlying processes, and given the pivotal role of podocyte injury in proteinuria, we examined podocyte changes in DN mice using electron microscopy. The results revealed significantly greater fusion and disruption of podocyte foot processes and podocyte loss in the db/db + Ad-β-gal group compared to the db/m + Ad-β-gal and db/m + Ad-Renalase groups. When Renalase adenovirus was injected into the tail vein of the db/db + Ad-Renalase group, these lesions significantly improved (Figure 3a and b). Immunohistochemical analysis indicated that the expression of podocin and nephrin, marker proteins of podocytes, was significantly reduced in the db/db + Ad-β-gal group compared to the db/m groups; however, this change was notably relieved when Renalase was overexpressed in the db/db + Ad-Renalase group (Figure 3c). These findings suggest that Renalase can ameliorate podocytopathy in DN, potentially contributing to its ability to minimize the progression of DN.

### 3.4 Reduction of podocyte damage from Renalase may be related to the reduction of apoptosis and oxidative stress

**Figure 4:**
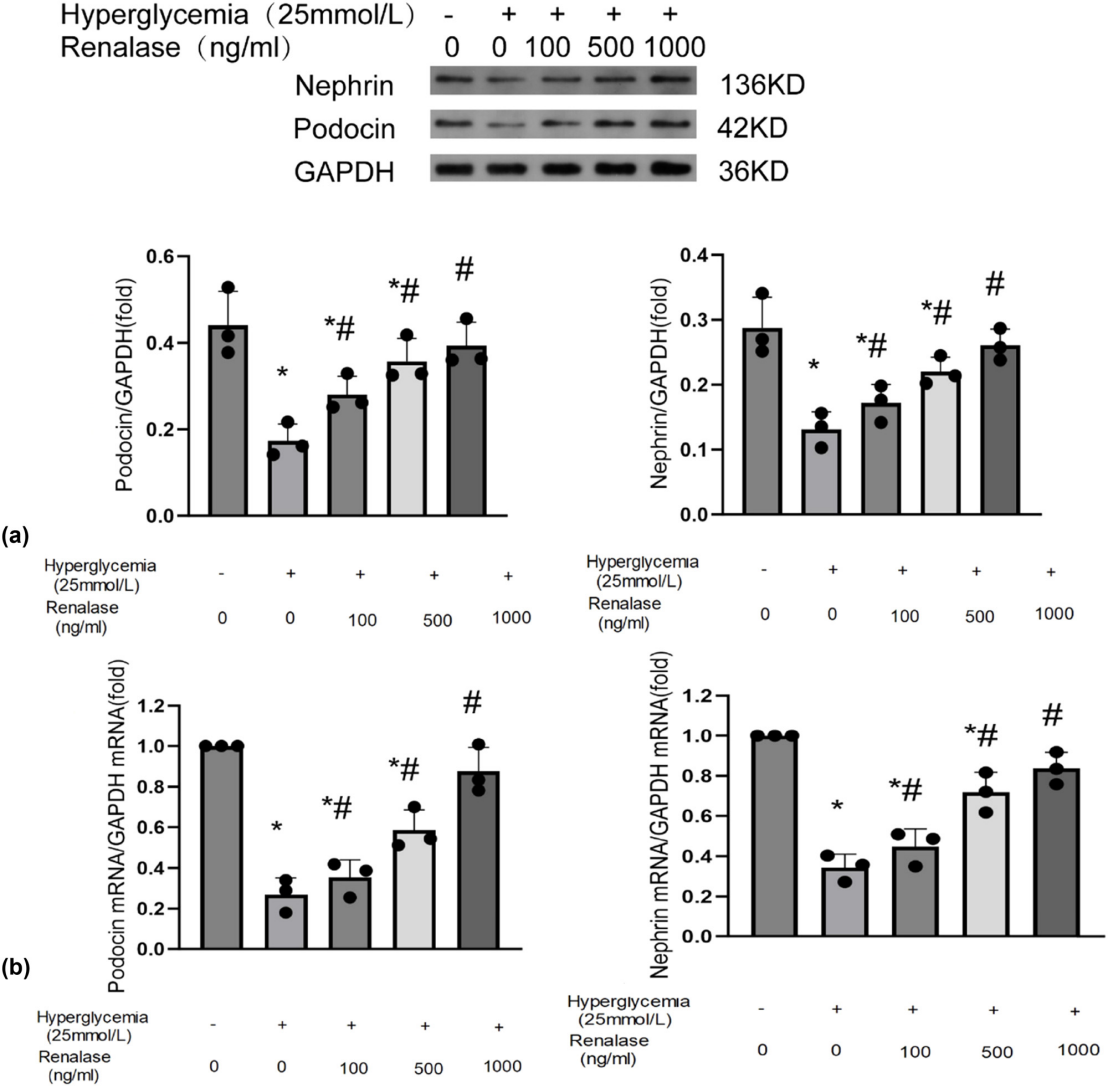
Renalase reduced podocyte injury *in vitro*. MPC5 were treated with different concentrations of glucose (5 and 25 mmol/L) and Renalase (100, 500, 1,000 ng/mL) for 48 h. Western blotting (a) and qT-PCR (b) revealed Renalase restored down-regulated podocin and nephrin expression to some extent in a dose-depended manner. Results are presented as percentages of control values after normalization to GADPH and are the mean ± SD of three independent experiments. *p < 0.05, compared with control groups; ^#^ *p* < 0.05, compared with HG groups.

**Figure 5:**
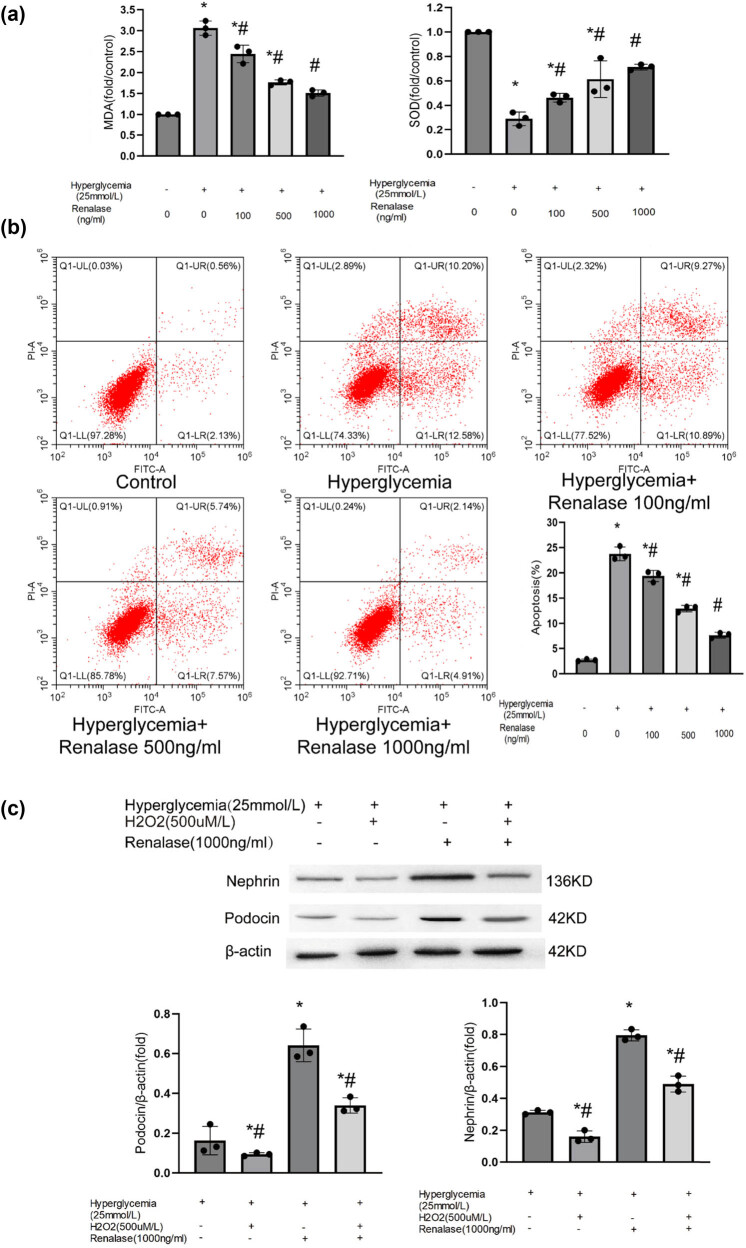

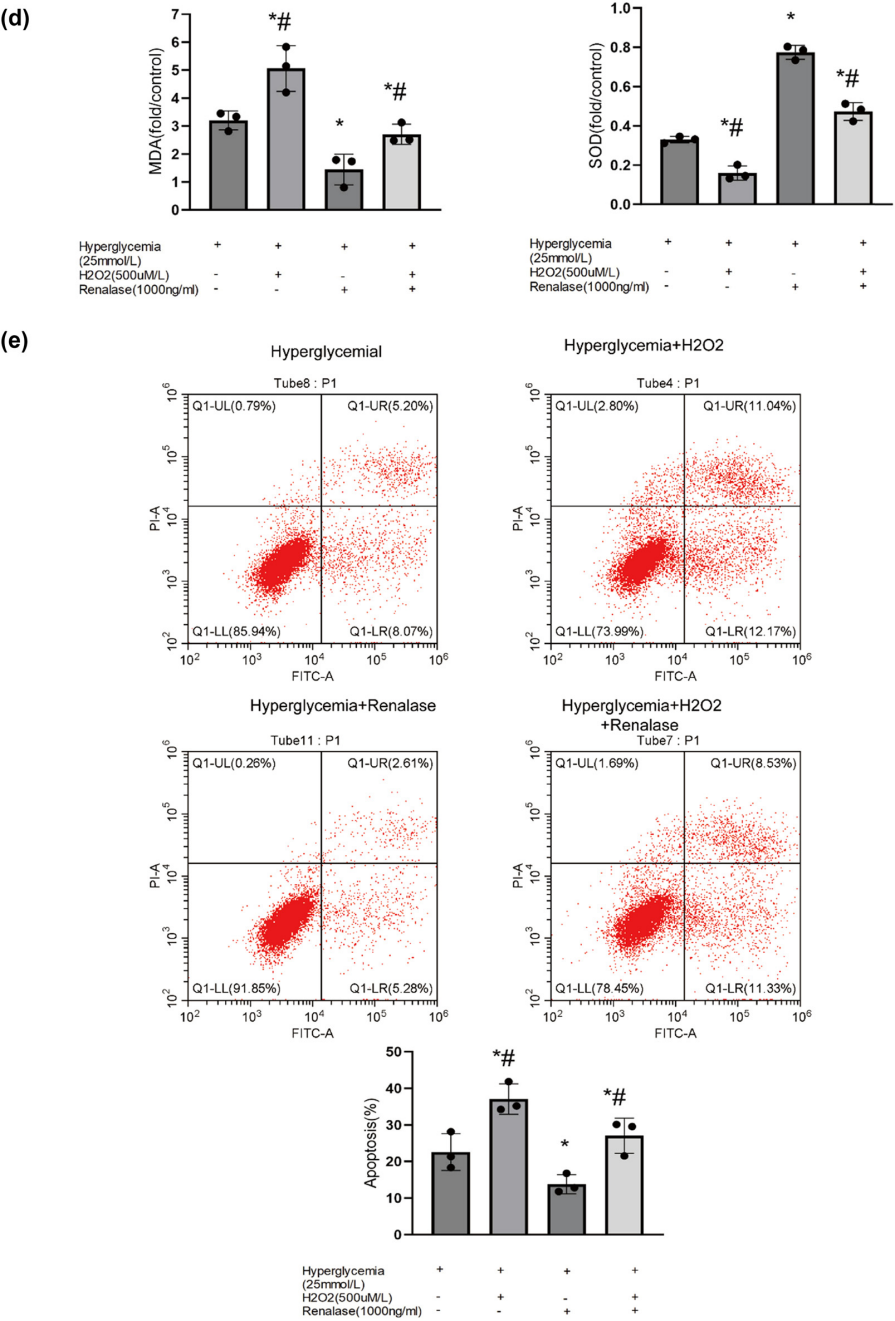
Renalase blocks HG-mediated oxidative stress and apoptosis in podocyte. MPC5 were treated with different concentrations of glucose (5 and 25 mmol/L) and Renalase (100, 500, 1,000 ng/mL) for 48 h. (a) Renalase (100, 500, and 1,000 ng/mL) abolished HG inducing the increased expression of MDA and preserved expression of SOD in a dose-dependent manner; (b) Renalase (100, 500, and 1,000 ng/mL) abolished HG inducing apoptosis in a dose-dependent manner. Results are presented as the mean ± SD of three independent experiments. *p < 0.05, compared with control groups; ^#^ *p* < 0.05, compared with HG groups. (c–e) MPC5 cells were incubated with HG (25 mmol/L) in the absence or presence of H_2_O_2_ (500 µM/L) and Renalase (1,000 ng/mL) for 48 h. It showed that the expression of nephrin and podocin further reduced (c) and oxidative stress (d) and apoptosis (e) further increased in HG + H_2_O_2_ group compared with HG group. Compared with HG + Renalase group, the expressions of nephrin and podocin decreased (c), while oxidative stress (d) and apoptosis (e) increased significantly in HG + H_2_O_2_ + Renalase group. *p < 0.05, compared with HG groups; ^#^ *p* < 0.05, compared with HG + Renalase groups.

We further investigated the potential processes by which Renalase alleviates podocyte injury *in vitro*. We treatedMPC5 with different concentrations of glucose and Renalase. Consistent with *in vivo* findings, the expression of podocin and nephrin was down-regulated after induction of HG. However, simultaneous treatment with Renalase restored podocin and nephrin expression at both the protein (Figure 4a) and mRNA levels (Figure 4b) in a dose-dependent manner. To elucidate the molecular processes, considering the crucial role of oxidative stress and apoptosis in podocyte injury, we assessed indicators related to oxidative stress and apoptosis. Renalase reduced levels of MDA and enhanced SOD levels in a dose-dependent manner compared to the HG group (Figure 5a). Additionally, apoptosis was significantly reduced when cells were incubated with Renalase (Figure 5b). These results suggest that Renalase may mitigate proteinuria in DN by inhibiting oxidative stress and apoptosis in podocytes. Further, based on previous research [18], MPC5 cells were incubated with HG (25 mmol/L) in the absence or presence of H_2_O_2_ (500 µM/L) and Renalase (1,000 ng/mL) for 48 h to explore its podocyte protection activity. Compared with the HG group, oxidative stress and apoptosis were exacerbated in the HG + H_2_O_2_ group, and the expressions of nephrin and podocin were further decreased (Figure 5c–e). This suggests that H_2_O_2_ can induce oxidative stress and apoptosis, exacerbating podocyte injury caused by HG. Compared with the HG + Renalase group, oxidative stress and apoptosis levels increased, and the expressions of nephrin and podocin significantly decreased in the HG + H_2_O_2_ + Renalase group, indicating that the protective effect of Renalase on podocytes was substantially negated by the increased oxidative stress and apoptosis induced by H_2_O_2_. These findings further validate that inhibiting oxidative stress and apoptosis is a key process by which Renalase can alleviate podocyte injury.

## 4 Discussion

DN is the primary cause of morbidity and mortality among diabetes patients and is also the leading cause of ESRD globally. While renin-angiotensin-aldosterone system (RAAS) blockades, blood pressure and glucose regulation, and smoking cessation all play roles in preventing the development and progression of DN; currently, there are no effective drugs available to halt its progression [19]. The pathogenesis of DN involves changes in renal hemodynamics, oxidative stress, inflammation, hypoxia, and hyperactivity of the RAAS, among which renal fibrosis is a crucial factor [19]. Previous studies have indicated that Renalase can reduce oxidative stress in chronic kidney diseases (CKD) and AKI, and our findings suggest that Renalase can also delay renal interstitial fibrosis [17,18,20]. Our research focused on the role of Renalase in DN, revealing that Renalase expression was diminished in db/db mice. However, when Renalase was overexpressed *in vivo*, kidney lesions improved significantly. Proteinuria, an emblematic indicator of DN, is associated with the advancement of kidney disease and the increase of cardiovascular events [19]. Following the overexpression of Renalase, urinary protein levels significantly decreased in db/db mice, corroborating earlier findings [16]. Proteinuria, often resulting from podocyte detachment and apoptosis or injury, is a central contributor to DN and manifests as hypertrophy, cell flattening, and foot process effacement [21]. Our observations indicate that Renalase can mitigate podocyte damage both *in vivo* and *in vitro*. It appears that this protective effect may be linked to the inhibition of oxidative stress and apoptosis, as demonstrated *in vitro*; however, when oxidative stress and apoptosis were induced by H_2_O_2_, the protective effect of Renalase on podocytes was diminished. No significant effects of Renalase on overall renal function were observed in this study, possibly due to the specific animal models used. It is important to note that early diabetic renal damage is prevalent in db/db mice, but renal function is not severely compromised [22].

By interacting with its plasma membrane receptor, Renalase exerts its cytoprotective effects without metabolizing catecholamines [23]. Research has established Renalase’s protective roles in several organs, including the cardiovascular [13], liver [24], and pancreas [25] systems. Additionally, Renalase has demonstrated a definitive protective effect on the kidneys. It has been shown to protect against the progression of CKD by inhibiting renal interstitial fibrosis [17,18]. By mitigating oxidative stress, Renalase also protects against renal ischemia-reperfusion injury as well as cisplatin-induced AKI both *in vitro* and *in vivo* [15]. Similarly, our findings indicate that Renalase provides protection in DN and reduces urinary protein, aligning with previous studies [16,26]. The progression of urinary protein due to podocyte injury is a significant factor in DN [8]. Podocyte injury in DN involves various factors including oxidative stress, inflammation, mitochondrial damage, autophagy, and other molecular signaling processes [21,27]. In this study, we observed significant podocytopathy in the db/db group compared to the db/m group, characterized by fusion of foot processes, loss of podocytes, and prominent urinary protein. Overexpression of Renalase in the db/db + Ad-Renalase group markedly reduced podocytopathy and significantly decreased urinary protein levels. These findings suggest that Renalase may reduce urinary protein by decreasing podocyte loss and thus delay the progression of DN *in vivo*. Additionally, the expression of the podocyte marker proteins, nephrin and podocin, decreased under high glucose stimulation in cultured podocytes, indicating that podocyte damage occurred, alongside increased oxidative stress and evident apoptosis. When Renalase was co-cultured simultaneously, there was a partial recovery in podocyte health, and both oxidative stress and apoptosis decreased in a concentration-dependent manner. However, when H_2_O_2_ was used to activate oxidative stress and apoptosis, the protective effects of Renalase on podocytes were diminished. This confirms that inhibiting oxidative stress and apoptosis is a key process by which Renalase can mitigate podocyte injury. We demonstrate for the first time that Renalase may reduce podocyte injury by inhibiting oxidative stress and apoptosis *in vitro*. Our previous studies indicate that Renalase plays a protective role in the kidneys by inhibiting the extracellular regulated protein kinases (ERK) signaling pathway; thus, whether Renalase can also improve podocyte injury in DN by targeting the ERK pathway remains a subject for further experimental investigation.

However, this study has certain limitations. First, we postulate that Renalase can reduce urinary protein and delay the progression of DN. However, some patients with DN do not exhibit significantly abnormal urinary protein but do show progressive renal function deterioration [1,28], The pathogenesis in these patients may differ from the conventional understanding. Therefore, whether Renalase has a protective effect on patients without proteinuria in DN needs to be confirmed by further studies. Second, Renalase is predominantly secreted by renal tubular epithelial cells [17], whereas podocytes are renal glomerular epithelial cells. The process by which Renalase secreted from renal tubular epithelial cells impacts podocytes *in vivo* – whether through paracrine or other signaling systems – has not been established in this study and warrants further exploration. Third, recent studies have demonstrated that Renalase also exerts a protective effect on the pancreas. Furthermore, exogenous supplementation of Renalase has been shown to mitigate the severity of acute pancreatitis [25]. Additionally, Renalase deficiency has been found to impact the metabolism of pancreatic beta cells [29], abnormalities of which are strongly linked to type 1 diabetes. Therefore, in this study, while there was no discernible difference in blood sugar levels between the two groups, it remains unclear whether Renalase has an impact on the pancreas of type 2 diabetic mice and whether its protective effect on the kidneys of type 2 diabetic mice is associated with regulating pancreatic cell function. Finally, although many studies have confirmed that Renalase has various protective effects with a certain concentration dependence, some studies have found that Renalase can promote tumor growth to some extent [30]. Although we did not observe tumor phenomenon in mice in this study, this may be related to the relatively low concentration and a short observation period of Renalase. If Renalase is developed as a new drug, the optimal concentration of Renalase should be explored, which can exert the maximum protective effect without causing major side effects, which needs further research.

In conclusions, our results confirmed the protective effect of Renalase in DN, which may be related to the reduction of urinary protein by alleviating podocyte injury through inhibiting oxidative stress and apoptosis. It has been shown Renalase acts as an underlying suppressor of DN. Therefore, the complement of exogenous Renalase may be a prospective approach to slowing or preventing the evolution of DN. This study offers a theoretical foundation for the clinical investigation and development of Renalase as a novel pharmaceutical intervention to mitigate proteinuria in DN.
